# Paget’s disease: a review of the epidemiology, etiology, genetics, and treatment

**DOI:** 10.3389/fgene.2023.1131182

**Published:** 2023-04-26

**Authors:** Babajan Banaganapalli, Ibrahim Fallatah, Fai Alsubhi, Preetha Jayasheela Shetty, Zuhier Awan, Ramu Elango, Noor Ahmad Shaik

**Affiliations:** ^1^ Department of Genetic Medicine, Faculty of Medicine, King Abdulaziz University, Jeddah, Saudi Arabia; ^2^ Princess Al-Jawhara Al-Brahim Center of Excellence in Research of Hereditary Disorders, King Abdulaziz University, Jeddah, Saudi Arabia; ^3^ Department of Biomedical Sciences, College of Medicine, Gulf Medical University, Ajman, United Arab Emirates; ^4^ Department of Clinical Biochemistry, Faculty of Medicine, King Abdulaziz University, Jeddah, Saudi Arabia

**Keywords:** Paget’s disease of bone, genetics factors, environmental factors, osteoclast (OCs), SQSTM1

## Abstract

Paget’s disease of bone (PDB) is the second most prevalent metabolic bone disorder worldwide, with a prevalence rate of 1.5%–8.3%. It is characterized by localized areas of accelerated, disorganized, and excessive bone production and turnover. Typically, PDB develops in the later stages of life, particularly in the late 50s, and affects men more frequently than women. PDB is a complex disease influenced by both genetic and environmental factors. PDB has a complex genetic basis involving multiple genes, with SQSTM1 being the gene most frequently associated with its development. Mutations affecting the UBA domain of SQSTM1 have been detected in both familial and sporadic PDB cases, and these mutations are often associated with severe clinical expression. Germline mutations in other genes such as TNFRSF11A, ZNF687 and PFN1, have also been associated with the development of the disease. Genetic association studies have also uncovered several PDB predisposing risk genes contributing to the disease pathology and severity. Epigenetic modifications of genes involved in bone remodelling and regulation, including RANKL, OPG, HDAC2, DNMT1, and SQSTM1, have been implicated in the development and progression of Paget’s disease of bone, providing insight into the molecular basis of the disease and potential targets for therapeutic intervention. Although PDB has a tendency to cluster within families, the variable severity of the disease across family members, coupled with decreasing incidence rates, indicates that environmental factors may also play a role in the pathophysiology of PDB. The precise nature of these environmental triggers and how they interact with genetic determinants remain poorly understood. Fortunately, majority of PDB patients can achieve long-term remission with an intravenous infusion of aminobisphosphonates, such as zoledronic acid. In this review, we discuss aspects like clinical characteristics, genetic foundation, and latest updates in PDB research.

## Introduction

Paget’s disease of bone (PDB) is a chronic and progressive bone disease that is characterized by bone pain, deformities, and fractures. The term “osteitis deformans” was first coined by Sir James Paget, an English physician, in 1877 (Paget, 1877). However, evidence of the disease dates back to 3,000 years, as suggested by lesions resembling PDB found in dinosaur vertebrae from the late Paleozoic to the middle Mesozoic periods (Haridy et al., 2019; Gennari et al., 2022). PDB is characterized by abnormal activation of osteoclasts, which leads to improper bone resorption and compensatory osteogenic sclerosis (Tuck, 2020). The disease is associated with increased bone remodeling and mass, with abnormal osteoclast activity leading to increased metabolic osteolytic activity while osteoblasts produce bone normally (Alonso et al., 2017).

## Epidemiology of PDB

PDB is the second most prevalent disorder of bone remodeling, after osteoporosis, despite it being asymptomatic in many with variable late age of onset. The incidence of PDB varies depending on the population studied and the diagnostic criteria used, but it is generally considered to be a rare disease. The global prevalence of PDB ranges from 1.5% to 8.3%, highest in Europeans living in the United Kingdom, followed by Australia, New Zealand, North America, France, Germany, Spain and Italy. Conversely, it appears rare among Scandinavians, Africans, Asians, and non-European immigrants living in Europe (Vallet and Ralston, 2016; Nebot Valenzuela and Pietschmann, 2017; Abdulla et al., 2018). Among Middle East Arabians in southern Israel revealed a 1% prevalence of PDB, comparable to southern Europe. In Saudi Arabia, Iran, Iraq, and Turkey, few isolated case reports of PDB have been recorded (Alshaikh et al., 2011; Merashli and Jawad, 2015; Michou and Orcel, 2019). The above epidemiological data confirms that there is marked geographical variation in the occurrence of PDB. But whether this is linked to genetic susceptibility of specific ethnic or racial population groups, and/or potential environmental influences like diet (mineral and vitamin deficiencies), lifestyle (tobacco smoking) exposure to the pollutants (lead), and an infection (paramyxovirus) is unclear.

### Clinical manifestations

**FIGURE 1:**
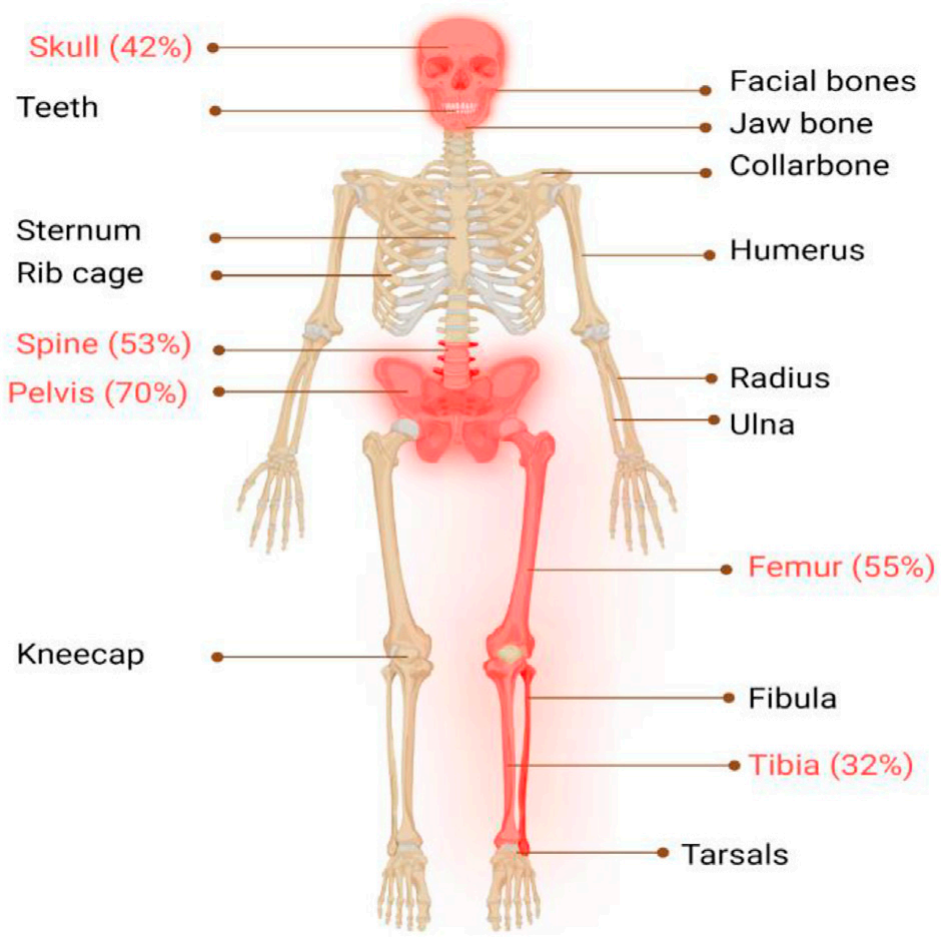
Illustrates the major skeletal locations affected by PDB: “The disorder predominantly affects the axial skeleton, with the highest incidence observed in the pelvis (70%), femur (55%), lumbar spine (53%), cranium (42%), and tibia (32%). To provide a visual representation of the most commonly affected areas in PDB, these locations are highlighted in red."

**FIGURE 2:**
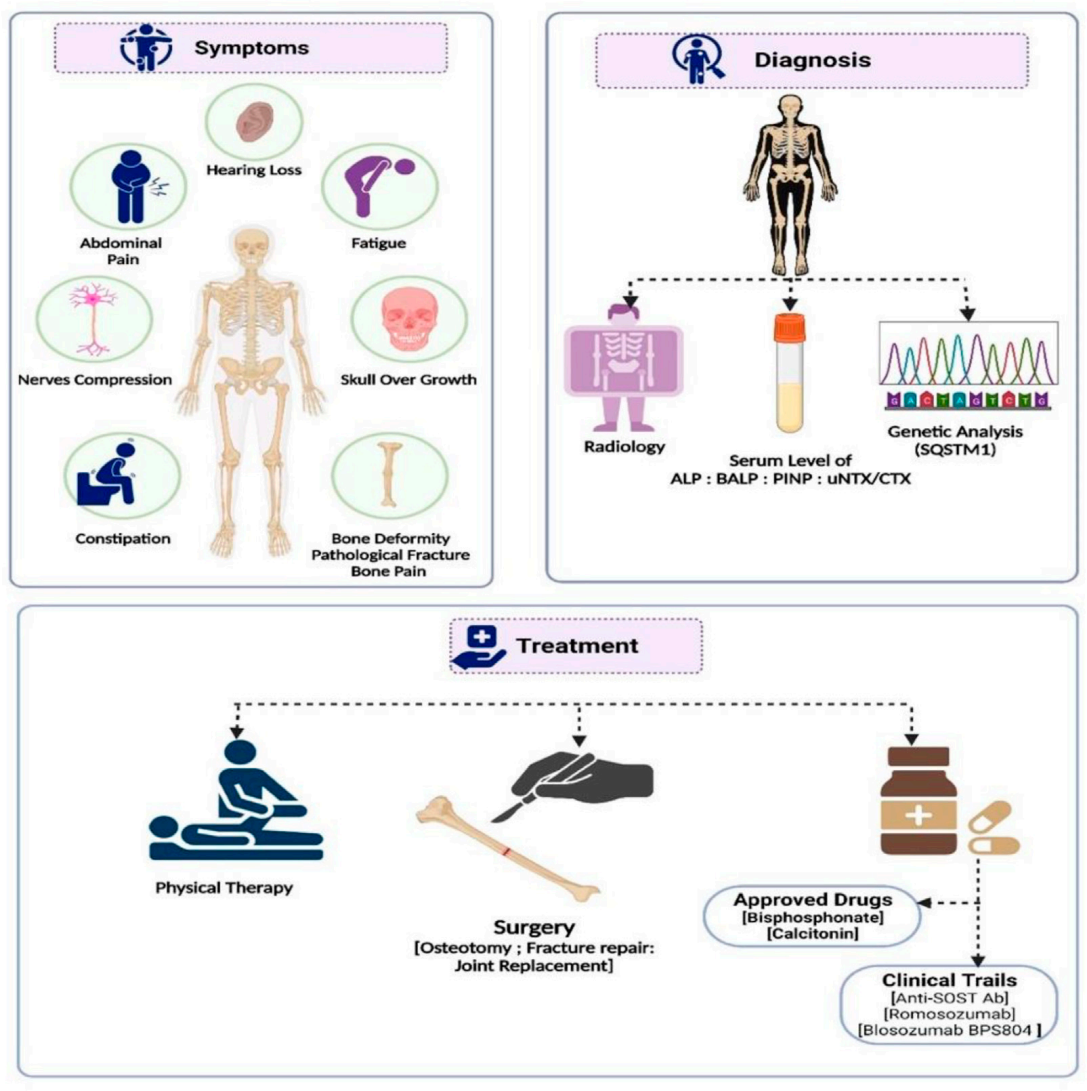
A summary of the diagnosis, management, and treatment approaches to Paget’s disease of bone.

The classical PDB usually appears at the age of forty and is rarely diagnosed before the age of fifty, with a slight male predominance (Vallet and Ralston, 2016). In around 70% of patients, PDB is asymptomatic and is typically discovered incidentally through elevated alkaline phosphatase (ALP) values (Kravets, 2018). When symptoms are present, the most frequent symptom is bone pain (73.8%), followed by morphological conditions (18.1%), hearing loss (7.9%), and pathological fractures (5.7%) (Ralston and Albagha, 2018; Michou and Orcel, 2019). Bone deformities, such as bowing of the legs, skull enlargement, and kyphosis, may also occur. Hearing loss, vision problems, and headaches can result from cranial nerve involvement. Increased blood flow to the bones can cause warmth and redness in affected areas. In rare cases, pathological fractures may occur. Leontiasis ossea, a condition in which the facial bones become deformed, can also occur. The most severe consequences of PDB include osteosarcomas and other sarcomas (chondrosarcoma, fibrosarcoma), though their incidence is less than 1% (Makaram and Ralston, 2021). In addition, PDB primarily affects the skeletal system, with certain regions being more prone to its impact than others (Makaram et al., 2020), as seen in Figure 1, the axial skeleton is the most commonly affected area (Figure 2).

### Biochemical assessment of PDB

Paget’s disease of bone (PDB) can be diagnosed through clinical, radiographic, and biochemical assessments. Biochemical assessment involves measuring serum alkaline phosphatase (ALP) and urinary N-telopeptide (NTx) levels. ALP is an enzyme produced by osteoblasts, responsible for bone formation. In PDB, bone formation increases, leading to elevated ALP levels (Gennari et al., 2019). However, ALP levels can also be elevated in other conditions, such as liver disease or during pregnancy, making additional tests necessary for diagnosis confirmation (Seton, 2013; Gennari et al., 2019). In contrast, NTx is a specific marker of bone resorption, where osteoclasts break down bone tissue. In PDB, there is an increase in both bone formation and resorption, leading to elevated NTx levels. However, NTx levels can be affected by other factors, such as age, gender, and menopausal status, necessitating interpretation in the context of clinical findings (Delmas, 1999). Additional tests, including bone scans and imaging studies, may also be useful in PDB diagnosis to assess the extent of the disease and risk of fractures. Serum procollagen type 1 amino-terminal peptide (P1NP), osteocalcin, and bone-specific ALP (BALP) are more sensitive biochemical markers for bone production, while peptides of the collagen type I cross-linking domains, including NTx or CTX, are more specific for bone resorption. Despite the characteristic elevations in ALP and NTx levels in individuals with PDB, these markers are not specific to the condition (Cook and Wall, 2021). Thus, diagnostic confirmation and treatment response monitoring require interpretation in the context of clinical findings and other diagnostic tests (Alonso et al., 2017).

### Radiological presentations in PDB

Radiographic changes can help diagnose Paget’s disease of bone (PDB). Indicators of increased bone resorption include a decrease in bone density, wedge-shaped bone resorption in long bones, and significant osteolytic regions in the skull. Early-stage PDB may show primarily lytic lesions, while older lesions tend to have a mixed sclerotic and lytic appearance. Late-stage PDB is characterized by sclerotic lesions, enlarged and distorted bones, and distinct radiographic patterns (Gennari et al., 2019; Ralston et al., 2019). The damaged bone enlargement in diameter is a distinguishing feature of PDB. A set of plain X-rays and bone scintigraphy are used to evaluate PDB, but bone scintigraphy may be negative in some cases. The presence of aberrant trabeculae, irregular cementation lines, increased vascularity, and an increase in the number and size of osteoclasts are the most distinctive findings (Alonso et al., 2017). Since genetic or bone biomarkers alone may lack sensitivity, combining many diagnostic markers is preferable to detect PDB at early stages and in asymptomatic cases. The PDB phenotype may be detected more accurately by integrating a screen for SQSTM1 gene mutations, followed by either a gene panel or a combination of genetic and biochemical tests (Guay-Bélanger et al., 2016). Approximately 70% of PDB patients have no symptoms, making early diagnosis challenging (Merchant et al., 2009). However, 15%–40% of patients have a positive family history, and first-degree relatives have a higher risk of developing PDB. Screening with a serum alkaline phosphatase test every two to 3 years is recommended for at-risk family members (Seton, 2013). Paget’s disease can lead to complications such as bone deformities, fractures, osteoarthritis, and an increased risk of developing bone cancer (Sabharwal et al., 2014). For instance, patients with PDB have an elevated chance of developing osteosarcoma, and despite its rarity (0.3% of PDB individuals), vast majority of osteosarcomas (OS) diagnosed in adulthood occur in patients with PDB. Similarly, there have been observation of families in which PDB is accompanied by giant cell tumours (Makaram and Ralston, 2021). Early detection and treatment are crucial in preventing these complications and improving patient outcomes. Radiographs should be taken to confirm the diagnosis (Ralston et al., 2019), and targeted genetic testing can be offered for at-risk family members (Seton, 2013; Guay-Bélanger et al., 2016).

### Etiopathogenesis

The primary cause of PDB is believed to be the abnormal activation of osteoclasts, leading to improper bone resorption and compensatory osteogenic sclerosis. This results in increased bone remodeling and mass, with the osteoclasts being larger in size, number, and with more nuclei compared to normal cells (Albagha, 2015; Gennari et al., 2019). This increased metabolic osteolytic activity leads to bone destruction, but the normal osteoblasts continue to produce new bone. The result is structurally abnormal and weakened bones that are prone to fractures and deformities (Gennari et al., 2019). It is worth noting that while the activation of osteoclasts is the primary cause of PDB, the etiology of this activation is not yet fully understood. However, PDB is largely considered a multifactorial disease due to the combination of genetic and environmental factors contribute to its disease pathology.

### Molecular factors in PDB

Normal adult skeleton remodeling involves osteoclasts destroying bone and osteoblasts generating new bone tissue at locations of past bone resorption (Kenkre and Bassett, 2018). The remodeling cycle is highly controlled and stereotypical, with five overlapping phases of activation, resorption, reversal, formation, and termination in cortical and trabecular bone respectively, within a period of 120–200 days. Osteocytes orchestrate the bone remodeling process by controlling osteoclast and osteoblast differentiation, and hence bone resorption and synthesis (Bellido et al., 2014). Several genes have been linked to osteoclast differentiation and activation which in turn leads to bone resorption (Albagha, 2015; Chen et al., 2018; Ralston and Albagha, 2018).

**FIGURE 3:**
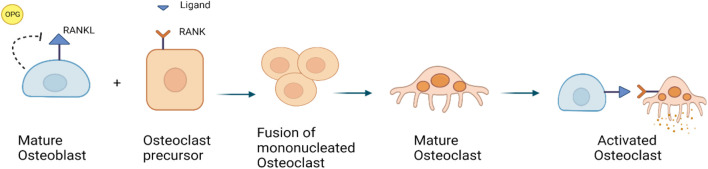
RANKL/RANK/OPG signaling pathway: RANKL is a receptor activator of nuclear factor kappa-B ligand, that is expressed by osteoblasts. OPG is also expressed by osteoblasts, it can bind to and inhibit RANKL and act as a protector against bone loss.

At the cellular level, normal bone remodeling is regulated by the receptor activator of nuclear factor kappa B (NF-kB) ligand (RANKL)/receptor activator of NF-kB (RANK)/Osteoprotegerin (OPG) system, which also controls the production and activity of osteoclasts (Kenkre and Bassett, 2018; Bolamperti et al., 2022). OPG, a decoy receptor, binds to RANKL to prevent RANK binding. Thereby, OPG suppresses the differentiation of osteoclasts. RANKL is expressed in the marrow stroma and on osteoblasts, when RANK binds on osteoclast precursors, it increases osteoclast proliferation and differentiation. It leads to the activation of a variety of downstream signaling pathways, including the nuclear factor kB (NF-kB), protein kinase B, c-jun N-terminal kinase, p38 mitogen-activated protein kinase and ERK pathways (Figure 3) (Tekkesin et al., 2011; Bellido et al., 2014).

### Genetic factors in PDB

Multiple lines of evidence suggest that inherited factors play an important role in PDB. The cumulative risk for developing PDB for a first-degree relative of an individual with PDB is estimated to be 9%, compared to 2% for those with unaffected relatives (Gennari et al., 2019). Recent evidence suggests that up to 40% of the PDB patients have a positive family history of PDB related symptoms or diseases. (Morales-Piga et al., 1995; Gennari et al., 2019; Gennari et al., 2022). At least one-third of patients have an autosomal dominant inheritance pattern with higher penetrance with age, risk in first-degree relatives will be as high as 50% (Ralston and Albagha, 2018; Gennari et al., 2019; Gennari et al., 2022). Though ∼40% of PDB patients had a positive family history for the illness, exclusion of undiagnosed, asymptomatic individuals makes it hard to determine the true incidence of familial disease (Morales-Piga et al., 1995). Intriguingly, the proportion of patients with a family history varies significantly between countries, ranging from approximately 5% in the Netherlands to 50% in the French-Canadian population. Between 12% and 15% of PDB patients in the United Kingdom and Italy have a family history (Merlotti et al., 2005; Langston et al., 2010). The marked ethnic differences in PDB prevalence support a strong genetic basis (Morales-Piga et al., 1995; Hocking et al., 2000; Morissette et al., 2006).

The genetic basis of PDB is complex and involves multiple genes. One of the most commonly implicated genes is SQSTM1, which encodes a multifunctional p62 protein (Shaik et al., 2021). Mutations in this gene have been found in up to 50% of people with familial PDB (Appelman-Dijkstra and Papapoulos, 2018). The p62 protein plays a crucial role in autophagy, a process involved in removing damaged cellular components. When this function is compromised, it can lead to the accumulation of damaged proteins in bone cells. The mutations in the SQSTM1 gene are responsible for both sporadic (10%–15%) and familial (25%–40%) forms of PDB. Studies have shown that the Pro392Leu mutation is present in about 46% of familial PDB cases of French-Canadian origin from Quebec, while two more mutations were found in a family of predominantly British background. In both studies, the P392L mutation was identified in 16% and 8.9% of sporadic PDB patients (Hocking et al., 2002; Laurin et al., 2002). Although SQSTM1 mutations are typically heterozygous, rare instances of homozygosity have also been reported (Rea et al., 2009). Over 30 distinct missense or truncating SQSTM1 mutations have been detected in up to 50% of familial and 20% of sporadic PDB cases in diverse populations (Gennari et al., 2019). In Hungary, about 21.95% of PDB patients carry the common p. Pro392Leu mutation in the SQSTM1 gene (Donáth et al., 2021).

Genotype-phenotype correlation studies have suggested that patients with nonsense mutations causing partial translation of UBA domain, have more severe and extensive disease than patients with missense mutations (Gennari et al., 2010; Visconti et al., 2010). Missense mutations are mostly restricted to exons 7 (29.41%) and 8 (70.59%) of the SQSTM1 gene (Shaik et al., 2021). However, even among patients with the same mutation and within the same family, clinical heterogeneity is reported (Laurin et al., 2002) (Gennari et al., 2010). Also somatic mutations in the SQSTM1 gene have been reported in sporadic papillary osteosarcoma (Merchant et al., 2009). In around 5% of PDB patients, somatic mutation in SQSTM1 (P392L) was observed in monocyte lineage only. Interestingly, PDB patients with this somatic mutation had a milder bone phenotype than those with the same mutation as a germline mutation (Guay-Bélanger et al., 2015). In germline mutations, the defect is present in every cell, while somatic mutations are seen in a subset of cells only, leading to variable expression of normal and abnormal proteins with reduced function. This might explain the milder phenotype in the somatic mutation carriers. Although SQSTM1 mutations are detected in about 50% of familial cases from various countries, their occurrence is relatively low in sporadic cases. Genome-wide association studies have revealed seven unique potential loci that account for ∼13% of the family risk of PDB in SQSTM1-negative individuals (Albagha et al., 2010; Albagha et al., 2011; Database, 2022).

### Other PDB-associated genes

The genome wide scans have revealed several susceptibility loci for PDB and related syndromes (Makaram and Ralston, 2021; Gennari et al., 2022). Most of these gene loci are linked to osteoclast differentiation or function. These genetic loci are identified by few rare and common genetic variants, which collectively increase the PDB risk. Table 1 shows the list of different genetic loci and corresponding genes involved in the predisposition of individuals to PDB (Albagha et al., 2010; Albagha et al., 2011; Albagha, 2015). The *TNFRSF11A* gene mutations were first reported in isolated cases of PDB and other PDB-like illnesses, such as Familial expansile osteolysis (FEO) and Expansile skeletal hyperphosphatasia (ESH). Mutation of the *TNFRSF11A* gene revealed several insertions at the exon 1, resulting in the duplication of amino acid sequences in the RANK signal peptide (Hughes et al., 2000). Until now, many heterozygous in-frame tandem duplications of varied length, resulting in longer RANK signal peptide, have been reported, with the majority are related to uncommon PDB-like illnesses (Ralston and Taylor, 2019). This gene encodes a protein belonging to the TNF-receptor superfamily. This receptor can interact with many TRAF family members, thus activating NF-kappa B and MAPK8/JNK. This receptor is also a key osteoclast development mediator. The receptor activator of NFkB (RANK) is encoded by the *TIFRSF11A* gene.

**TABLE 1:** PDB-predisposing risk genes with chromosomal location, encoded proteins, function, and diseases distinct from PDB (PDB-related disorders).

Chr.	Gene	Protein	Description/function	PDB-related disorders	References
5q35.3	*SQSTM1*	p62	This gene encodes a multifunctional protein that binds ubiquitin and activates NF-kB. Mutation in this gene causes sporadic and familial bone Paget disease.	**-**	Hocking et al. (2001), Laurin et al. (2001), Laurin et al. (2002)
18q21	*TNFRSF11A*	RANK	Greater NF-KB signaling activation *in vitro* correlates with higher disease severity *in vivo*.	Familial expansile osteolysis (FEO), Expansile skeletal hypophosphatasia (ESH)	Cody et al. (1997), Good et al. (2001), Nakatsuka et al. (2003)
1q21.3	*ZNF687*	C2H2 zinc finger protein	The protein that is encoded by this gene may have a significant function in the differentiation and development of bones.	PDB, pagetic osteosarcomas and in undifferentiated pagetic sarcoma	Divisato et al. (2016), Scotto di Carlo et al. (2020)
8q24.12	*TNFRSF11B*	OPG	Decoy receptor that regulates bone resorption negatively.	Juvenile PDB	Scotto di Carlo et al. (2020)
8q22	*DCSTAMP (TM7SF4)*	TM7SF4	Fusion of osteoclast precursors to develop mature osteoclasts with multiple nuclei.	**-**	Kukita et al. (2004), Yagi et al. (2005), Albagha et al. (2011)
10p13	*OPTN*	Optineurin	Key regulator of osteoclast survival and development. ∼60% increase the risk of developing the disease	**-**	Hocking et al. (2001)
1p13	*CSF1*	M-CSF	Primary controller of osteoclast survival and development	**-**	Tsurukai et al. (2000).
9p13.3	*VCP*	p97	Protein degradation, intracellular membrane fusion, DNA repair and replication, cell cycle control, and NF-kappa B pathway activation**.**	Inclusion body myopathy with PDB and frontotemporal dementia syndrome (IBMPFD)	Kovach et al. (2001), Watts et al. (2004)
6p21.31	*FKBP5*	Cis-trans prolyl isomerase	The encoded protein participates in the regulation of the immune system and fundamental physiological processes such as protein folding and transportation.	**-**	Bouwmeester et al. (2004), Lu et al. (2017)
6p21	*HLA*	HLA	PDB1 locus seem to play minor role in development of PDB	**-**	Fotino et al. (1977), Tilyard et al. (1982), Good et al. (2001)
2q36	*Unknown*	*Unknown*	The putative locus on chromosome 2q36 showed linkage under a heterogeneity model but not a homogeneity model.	**-**	Hocking et al. (2001)
7q33	*NUP205*	nucleoporin 205 kDa	It encodes nucleoporin 205 kDa, a transport-related component of the nuclear pore. However, its function in bone remains uncertain.	**-**	Lin et al. (2004).
15q24	*PML*	Promyelocytic leukemia protein	Osteoclast differentiation, survival, and resorption (mice)	**-**	Lin et al. (2004).
14q32	*RIN3*	Rab and Ras interactor 3	Vesicular trafficking, especially expressed in osteoclasts	-	Saito et al. (2002), Kajiho et al. (2003), Albagha et al. (2011)

The *ZNF687* gene encodes for C2H2 zinc finger protein that may play a role in bone differentiation and development. Mutation in *ZNF687* gene was identified for the first time in large Italian family many affected family members by whole exome analysis, followed by other studies confirms it as causal gene for PDB (Divisato et al., 2016; Scotto di Carlo et al., 2020). The *ZNF687* gene mutation was also associated with polyostotic PDB, pagetic osteosarcomas and in undifferentiated pagetic sarcoma tissue as well (Scotto di Carlo et al., 2020). For instance, minority of PDB patients develop malignant giant cell tumors (GCTs) of the bone (PDB/GCTs) with an early onset (Wei et al., 2021). ZNF687 is the only gene currently proven to cause PDB/GCT; however, Profilin 1 gene have recently been identified as the cause of early-onset Paget’s disease of bone with GCT in Italian and Chinese patients (Wei et al., 2021). Meanwhile, individuals with mutations in the *ZNF687* gene experience severe PDB at multiple sites. *In vitro* studies have revealed that *ZNF687* mutant osteoclasts can have up to 150 nuclei, a finding unique to PDB patients with *ZNF687* mutations (Divisato et al., 2016; Scotto di Carlo et al., 2020). Knock-in mouse model of ZNF687 mutation supports the crucial role it plays in the PDB development and its potential oncogenic property by having high incidence of hepatocellular carcinomas (Russo et al., 2023).

Complete *TNFRSF11B* gene deletion is associated with JPD (Juvenile Paget Disease) (Scotto di Carlo et al., 2020; Naot et al., 2014). Also, partial *TNFRSF11B* gene resulting in loss of a conserved aspartate residue at codon 192 was also identified (Middleton-Hardie et al., 2006). Nevertheless, *TNFRSF11B* mutations have not yet been discovered in classical PDB, and the 8q24 locus did not emerge as a susceptibility gene in GWAS. There is some indication that common polymorphisms at the *TNFRSF11B* gene may predispose women, but not men, to classical PDB; however, this must be validated by a large-scale investigation (Solomon, 1979; Beyens et al., 2007). This gene, encodes Osteoprotegerin (OPG) protein, belongs to the TNF-receptor superfamily; an osteoblast-secreted decoy receptor that regulates bone resorption negatively. Also, this protein controls osteoclast development and function by inhibiting the stimulatory effects of RANKL on osteoclast differentiation (Makaram and Ralston, 2021; Gennari et al., 2022). The mutant OPG is unable to block osteoclastic resorption in a bone culture system, demonstrating that it is a loss-of-function mutation (Chong et al., 2003).

The *DCSTAMP* gene (Dendrocyte Expressed Seven Transmembrane Protein, also known as TM7SF4), encodes for a seven-pass transmembrane protein and is expressed on cells that is involved in osteoclastogenesis, immunological activity, and myeloid differentiation. In addition, the fusing of osteoclast precursors into mature osteoclasts is facilitated by the *DCSTAMP* protein. During the osteoclast formation, expression of *DCSTAMP* is crucial. Variants in the genes that predispose to PDB may increase *DCSTAMP* expression, leading to the formation of massive, multinucleated, granulocytic osteoclasts (Kukita et al., 2004; Yagi et al., 2005; Albagha et al., 2011).

The *OPTN* gene encodes the protein optineurin, which has coiled-coil structures; highly expressed cytoplasmic protein with many physiological activities, including NFB signaling, autophagy, and innate immunity (Zhu et al., 2007; Wild et al., 2011). Previous investigations have found a negative regulatory function for *OPTN* in osteoclast formation by altering *in vitro* and *in vivo* NF-kB and interferon signaling in mouse models (Obaid et al., 2015) (Silva et al., 2018a; Silva et al., 2018b).

The chromosome 1p13 hosts a recombination hotspot in the vicinity of the CSF1 gene. Notably, CSF1 exclusively encodes M-CSF, a cytokine that plays a pivotal role in regulating osteoclastogenesis and modulating the activity and survival of macrophages (Tsurukai et al., 2000; Bouyer et al., 2007). It is a strong candidate for PDB susceptibility based on its function (Tsurukai et al., 2000). The function of *CSF1* in the etiology of PDB is supported by the observation that individuals with PDB have elevated blood levels of M-CSF (Neale et al., 2002). Variations in this gene that predispose to PDB is still unknown, although it is hypothesized that they may cause PDB via enhancing osteoclast production through CSF1 activity. A predisposing variation for PDB in CSF1 was found by a GWAS analysis (Albagha et al., 2010) and confirmed by a follow-up study the same group (Albagha et al., 2011). In parallel, a missense mutation in the *CSF1* gene was identified in JPD (Donáth et al., 2015). Moreover, the SNPs linked with PDB are situated upstream of the gene in a region rich in regulatory elements, suggesting that their influence on control of *CSF1* expression; however, the precise mechanism by which these variations predispose to PDB is yet to be determined (Neale et al., 2002).

*VCP* gene, encodes valosin-containing protein, which is a member of the AAA (+) ATPase family of chaperone-like proteins. It is a multifunctional protein involved in several intracellular processes including NF-KB signaling, DNA repair, and autophagy. Mutations in the *VCP* gene were discovered as the cause of the autosomal dominant Inclusion Body Myopathy with PDB and Frontotemporal Dementia (IBMPFD), characterized by skeletal defects identical to the classical PDB (Kovach et al., 2001; Watts et al., 2004; Columbres et al., 2023)

The *VCP* mutational effects include a modulatory influence on the NF-KB signaling pathway due to *VCP’s* involvement in the degradation of phosphorylated IkB (Woodman, 2003; Tresse et al., 2010). In addition, there is evidence that protein-coding mutations of *VCP* may arise infrequently in people with classical PDB who lack other components of IBMPFD syndrome; however, there is no conclusive data that common mutations at the *VCP* gene make the individuals susceptible to PDB (Albagha et al., 2014). Furthermore, syndromic IBMPFD linked with *VCP* mutations has been categorized as one of the multisystem proteinopathies, in which neurological and muscular abnormalities sometimes accompany the PDB (Taylor, 2015). Moreover, Myopathy occurs in up to 90% of IBMPFD families, whereas PDB and dementia have been identified in 43% and 37% of cases, respectively (Ralston and Taylor, 2019).

Another gene reported to be associated with the PDB is *FKBP5* gene. Mutation in a Chinese family with PDB and supported by mutant mouse model. The functional study of the mutant mouse shows hyperresponsive osteoclast precursor cells to RANK with significantly high bone resorbing function (Liu and Zhang, 2015). The *FKBP5* gene is a member of the immunophilin protein family, which is involved in immunoregulation and in fundamental physiological processes including protein folding and transport (Bouwmeester et al., 2004; Lu et al., 2017) (Zheng et al., 2021).

### Epigenetics in PDB

In the last two to three decades, researchers have revealed that epigenetic modifications can influence the development of Paget’s disease (Leach et al., 2001; Galson and Roodman, 2014). Epigenetic alterations are chemical changes that occur in DNA and the proteins that bind to it without altering the actual DNA sequence. Environmental factors such as food, stress, pollutants, and infections can all influence these alterations, which can affect gene expression. Recent research has discovered various epigenetic changes that contribute to the development of PDB. For example, low DNA methylation of the RANKL gene promoter region was discovered in Paget’s disease patients compared to healthy controls, resulting in higher RANKL expression and associated bone resorption (Gennari et al., 2022). Similarly, increasing DNA methylation of the OPG gene promoter was linked to lower OPG expression, which accelerates bone resorption. Another important epigenetic component in PDB is histone modification. Histones are proteins that wrap DNA into nucleosomes, which are structural units. Histone acetylation and deacetylation are important gene expression regulators (Xu et al., 2020). Hypomethylation of histone H3 lysine 4 (H3K4me3) and hypermethylation of histone H3 lysine 9 (H3K9me3) have been linked to enhanced bone resorption. Factors like aging and viral infections are also known to play a role in PDB epigenetic alterations. Aging is a well-known risk factor for PDB, and studies have indicated that epigenetic changes associated with aging, such as increased DNA methylation, influence to the disease development (Saul and Kosinsky, 2021) and (Maatallah et al., 2021). PDB has also been related to viral infections, such as the measles virus, and studies have shown that the virus can modify the epigenetic landscape of bone cells, contributing to the disease development. DNA methylation and histone modification play important roles in bone resorption and creation, while environmental variables including aging and viral infections also contribute to epigenetic alterations (Diboun et al., 2021; Diboun et al., 2022). Therefore, epigenetic changes play a significant role in the development of Paget’s disease. With a greater understanding of these changes, researchers may be able to develop novel treatments that target the epigenetic abnormalities caused by Paget’s disease and aid in preventing the disease’s progression.

### Role of environmental factors

Environmental factors are frequently proposed as an explanation for the variability in Paget’s disease epidemiology. For instance, river water contamination by arsenic in a pesticide used to treat cotton bales may have contributed to the high frequency of PDB in Lancashire during the 1970s. Because the nuclear inclusion bodies in osteoclasts appear to reflect viral nucleocapsids of one of the paramyxoviruses, it has been hypothesized that viral infections may be one of the causes of PDB (Visconti et al., 2017). Other environmental factors, such as cigarette smoke and wood stove smoke during childhood, have also been linked to Paget’s disease. These exposures have decreased due to changes in lifestyle over the time (Audet et al., 2017). Lifestyle factors such as diet and physical activity also play a role in the development of PDB. A diet that is high in saturated fats and low in calcium and vitamin D can contribute to the development of the disease, as can a sedentary lifestyle. Exercise and a diet that is rich in calcium and vitamin D can help to prevent the development of PDB and slow the progression of the disease (Numan et al., 2019). Furthermore, experiments revealed a relationship between the SQSTM1 gene and exposure to cadmium and tobacco smoke condensates, in certain patients with non-familial PDB (Numan et al., 2019). Addressing these environmental factors could prevent PDB development and improve outcomes. However, more research is needed to establish the exact mechanisms by which environmental factors contribute to PDB development.

### Treatment of PDB

PDB does not have a specific treatment. However, some drugs can significantly improve the clinical symptoms and disease management. Bisphosphonates are the most used and FDA-approved drugs for treating PDB, and nitrogen-containing bisphosphonates have been found to be more effective than non-nitrogen-containing ones (Ralston et al., 2019). Patients with moderate-to-severe Paget’s disease of bone (PDB) have achieved successful outcomes with either a 6-month daily dose of alendronate (40 mg) or a 2-month daily dose of risedronate (30 mg). In approximately 60%–70% of cases, treatment normalized the ALP levels, and some patients were able to maintain biochemical remission for over a year (Gennari et al., 2009). Zoledronic acid is the most potent nitrogen-containing bisphosphonate and is the preferred choice for treating PDB due to its extended bone retention and intermittent dosing (Reid, 2016). However, its potential renal toxicity must be considered (Reid, 2016; Gennari et al., 2019). Oral alendronate or risedronate can also normalize alkaline phosphatase (ALP) levels in patients with moderate-to-severe PDB, but they may cause esophageal irritation and upper gastrointestinal tract (GI) discomfort. If patients cannot tolerate bisphosphonates, calcitonin is a safe, well-studied drug that can help lower the metabolic activity of pagetic bone and relieve GI discomfort (Reid, 2016). Surgery may be necessary in severe osteoarthritis cases but requires careful planning and preventative bisphosphonate medication. It is also crucial to tailor exercise programs and consume daily doses of vitamin D (400 IU) to maintain skeletal health (Ralston et al., 2019).

## Conclusion

PDB has long been regarded as the second most frequent disease of bone metabolism, after osteoporosis. However, a remarkable and ongoing drop in its frequency and severity in several nations, making PDB a rare form of bone disease. The cause of this phenomena is unknown, however the rapid change in countries with high PDB, such as the United Kingdom, suggests an environmental impact. Genetic studies have changed our understanding of the pathophysiology of PDB and uncovered novel genes and signaling networks that govern the development and activity of osteoclasts. Despite these advances, the environmental triggers of PDB remain poorly understood. It is unknown why PDB attacks some bones but not others. In addition to gaining a better biological understanding of PDB, it will be useful for elucidating disease processes in other illnesses that affect the skeleton in a targeted manner. Now, gene expression and epigenetics studies highlight that PDB shares some targetable biomarkers, however, the development of targeted molecular drugs for PDB patients is lacking. Therefore, molecular novel drug targets must be identified using a complete molecular diagnostic profile so personalized medicine and clinical management can be achieved in the coming decades.
